# Baseline data of marine debris in the Indonesia beaches

**DOI:** 10.1016/j.dib.2022.107871

**Published:** 2022-01-25

**Authors:** Ibnu Faizal, Zuzy Anna, Sanny T. Utami, Putri G. Mulyani, Noir P. Purba

**Affiliations:** a Department of Marine, Universitas Padjadjaran, Jatinangor, Jl. Ir. Sukarno Km. 21 UBR, West Java 40600, Indonesia; b Movement for The Ocean (MOCEAN), Bandung 40267, Indonesia; c Department of Fishery, Universitas Padjadjaran, Jatinangor, Jl. Ir. Sukarno Km. 21 UBR, West Java 40600, Indonesia; d College of Fisheries, Ocean University of China, Qingdao, China; e SDGs Center Universitas Padjadjaran, Jl. Dipati Ukur No.46, Lebakgede, Bandung, West Java 40132, Indonesia

**Keywords:** Marine environment, Beach pollution, Macro-debris, Baseline study, National action plan

## Abstract

This study was conducted around the beaches in Indonesia in order to investigate the level of pollution in the marine environment. Thirteen (13) locations in six (6) regions namely: Seribu Island, Banten, South Java, Biawak Islands, West Papua, and East Nusa making up a total length of 12.84 km of the area were studied. This investigation focused on the debris around isolated beaches, tourist attraction centers, fishing zones and marine protected areas (MPA). The method employed in this study was dependent on the international coastal cleanup form. The samples of debris collected and studied varied from the year 2013 to 2018 for a thorough investigation. The beach debris monitoring equipment revealed information about the distribution, abundance, types and, effects of marine debris on the ecosystem. Moreover, the study showed that the mass of debris collected within the areas listed weighted 1113.10 kg for 34,330 collected items. Also, the average density was noted to range between 1.43 and 5.11 items/m^2^. However, it was observed that plastic products constituted the highest percentage of the pollutants found in almost all the stations, with plastic bags being the most dominant.

## Specifications Table

**Table:** 

Subject	Environmental science
Specific subject area	Marine pollution: marine debris
Types of data	Table, Figure, Chart, Graph
How data were acquired	The data was collected by in-situ sampling using the International Coastal Cleanup form. The other instruments used were handheld GPS, rope transect, weighing scale, and trash bags.
Data format	Raw data and analyzed data
Description of data collection	The data collected comprised six categories of macro debris in several beaches. This data also contained the distribution, type, the weight of macro debris (>2.5 cm) from the surface beaches. The debris was collected from a transect in line within the range of 50-200 m.
Data source location	• Institution: Universitas Padjadjaran and KOMITMEN Research Group • Region: Bandung, West Java • Country: Indonesia • The data were collected in 13 locations, 6 regions, with 121 points sampling collected from 2013 to 2018 with Handheld GPS coordinates.
Data accessibility	Repository name: Mendeley Data Data identification number: 10.17632/r3y43cdd3x.2 Direct URL to data: https://data.mendeley.com/datasets/r3y43cdd3x/2
Related research article	Purba, N. P., Apriliani, I. M., Dewanti, L. P., Herawati, H., & Faizal, I. (2018). Distribution of macro debris at Pangandaran Beach, Indonesia. *World Scientific News, 103*, 144-156.

## Value of the Data

• The data collected in this study are very important because studies on marine debris distribution in Indonesia are relatively scarce.
• The data will be used by the government (both local and national) to mitigate debris deposition from various sources in the marine environment.
• The data will be of great advantage to researchers in order to conduct various studies such as the economic value, raising awareness and basis for modeling debris.
• Indonesia has been identified as the world's second-largest plastic polluter and it will require time-series data for policymakers and stakeholders to mitigate marine debris.
• Beaches are very important natural resources that could serve as tourist attractions, which could improve the standard of living both locally and nationally. However, one of the biggest problems identified in this area is the introduction of debris to the coastal environment.
• Debris on the beaches may come from any source, and the different beaches have been identified with different types of debris.

## 1 Data Description

Coastal areas have the potential to collect garbage from two main sources [1]. The first sources are wastes of natural origin such as river discharge and beach activities while the second type of wastes are man-made in nature such as vessels wastes discharged into the sea. Another source of man-made wastes are fishing activities [2]. In addition, the problems of pollution in the coastal region come from both within and outside (Boundary Transport) [3]. This research focused on the collection of debris in some coastal areas around Indonesia based on their abundance and geographic distribution. However, certain complex activities in these areas play a critical role in the distribution of garbage in the marine environment.

**Fig. 1:**
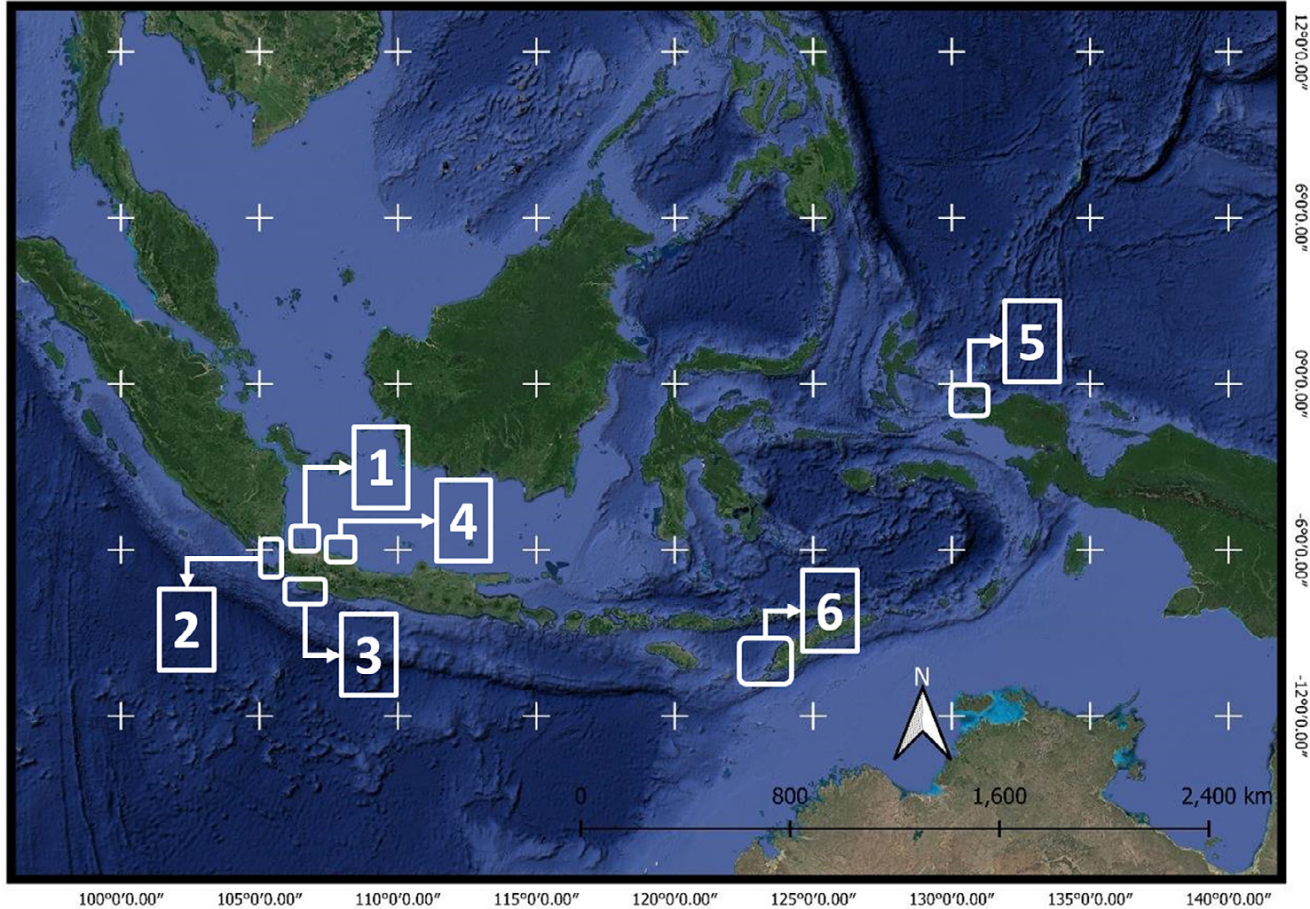
The study area is divided into 6 regions, (1) Seribu Islands, (2) Banten, (3) South Java, (4) Biawak Islands, (5) Misool, (6) the Savu Sea.

The dataset contained information about macro litter (>2.5 cm) acquired in 13 locations with a total of 121 sample points collected from 2013 to 2018 (Table 1; 10.17632/r3y43cdd3x.1)–the study sites are mostly located around Java Island, which is the most populated island in Indonesia (Fig. 1). The beach sites are characterized by tourism, coastal ecosystem, remote beaches, which include the small island, fishing, and marine protected areas (MPA). In general, site stations were situated in Seribu Islands (30 sites), Banten (17), South Java (22), Biawak Islands (9), West Papua (12), and East Nusa (29) (Fig. 1).

**Table 1:** Site sampling locations and characteristic.

Area	No.	Site	Year of Survey	Number of Station/Line	Characteristic
Seribu Island	1.	Pari Island	2014	4	Tourism activities
	2.	Harapan Island	2015	2	Residential
	3.	Untung Jawa Island	2016, 2017	22	Tourism activities
	4.	Kelor Island	2017	2	Tourism activities

Banten	5.	Handelum Island	2015	2	Marine Protected Areas
	6.	Panjang Island	2014	6	Fisheries activities
	7.	Tunda Island	2018	8	Residential

South Java	8.	Pangandaraan	2016, 2017	16	Tourism and fisheries activities
	9.	Palabuhan ratu	2016	8	Fisheries activities

Biawak Islands	10.	Biawak Island	2013, 2014	7	Remote beaches/island
	11.	Gosong Island	2016	2	Remote beaches/island (Atoll)

West Papua	12.	Misool Islands	2017	12	Remote beaches/island

East Nusa	13.	Savu Sea National Marine Conservation Area	2018	29	Marine Protected Areas

		**Total**		**121**

**Fig. 2:**
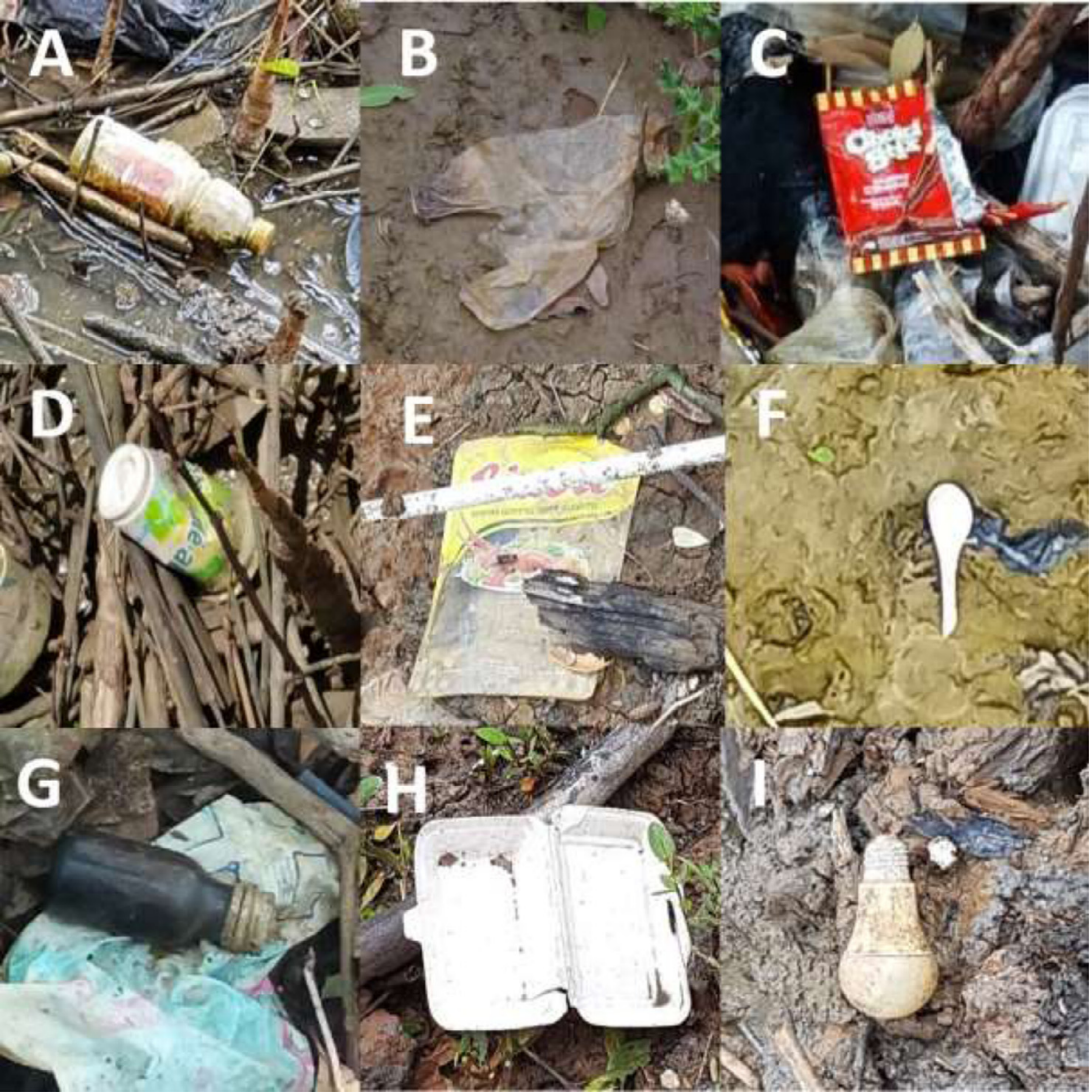
Sample of several marine debris found in this study: (A) Plastic bottles, (B) Grocery bags, (C) Plastic food wrapper, (D) Plastic cup, (E) Plastic wrapper, (F) Spoon, (G) Glass bottles, (H) Foam packaging, (I) Bulb.

Debris was collected from 121 stations in six areas, weighing around 1.1 tons with an average of 8.81 ± 0.08 kilos per 100 m and approximately 34,330 items collected from 12.84 km total length. An accumulated average of marine debris is 260 ± 2.51 items per 100 m of beach length were collected from all the stations. Both plastic and rubber items dominated the pollutants collected in each of the stations. Plastic bags, plastic bottles, foam, tires, and rope are samples of the most likely picked items in this category (Fig. 2).

**Fig. 3:**
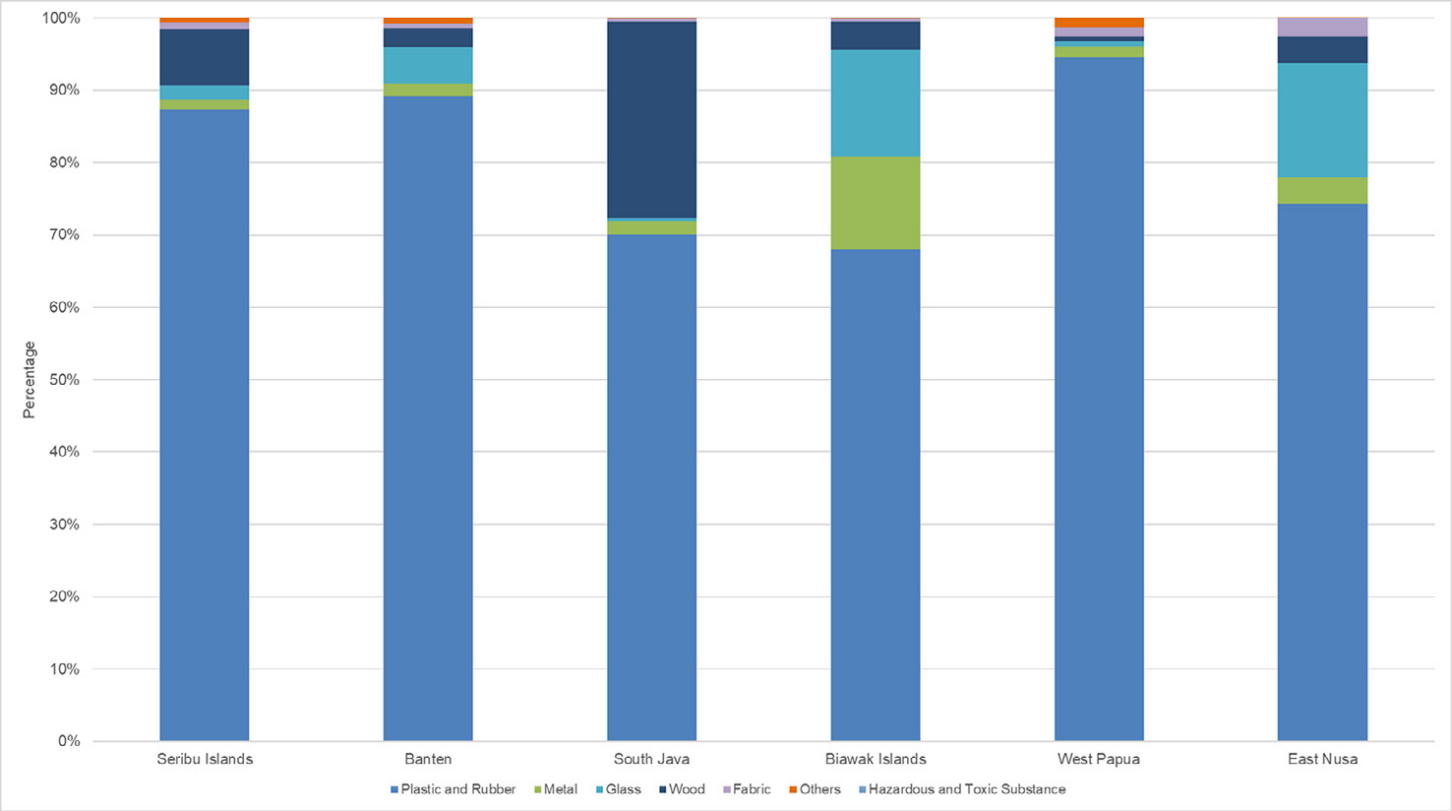
Distribution MD in Six Locations based on Six Categories. From seven categories of debris, dominant debris per types as follows: Plastics and rubbers are classified as Plastic bags (35.08%), Metals are classified as Cans (1.78%), Glasses are classified as Glass bottles (4.26%), Woods are classified as Cigarettes buds (15.87%), Fabrics are classified as Diapers and Tampons (1.07%), Others are Condoms (0.36%).

**Fig. 4:**
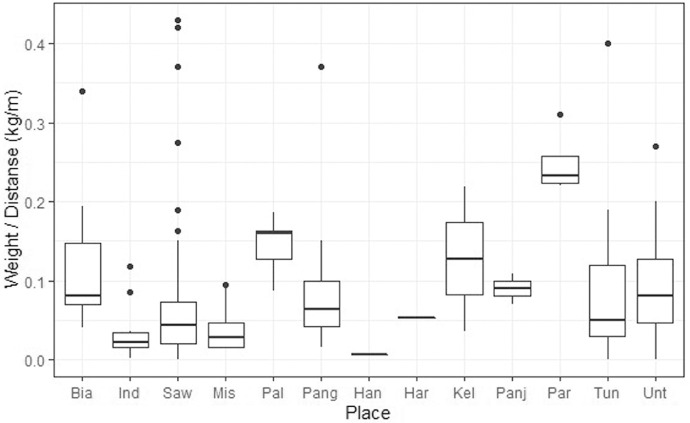
Average density (kg/m) per area. Many factors influence the great differences; the main factor affecting this study is the variation in the sample points across the number of stations investigated in each location.

The density of gathered debris from all the stations ranged between 0.002 and 0.43 kg/m^2^ with the highest varying from 0.01 to 18.71 items/m^2^. On average, South Java reported the highest marine debris from all observed stations with values of 5.11 Items/m^2^, and 0.096 kg/m^2^ (Figs. 3, 4).

**Fig. 5:**
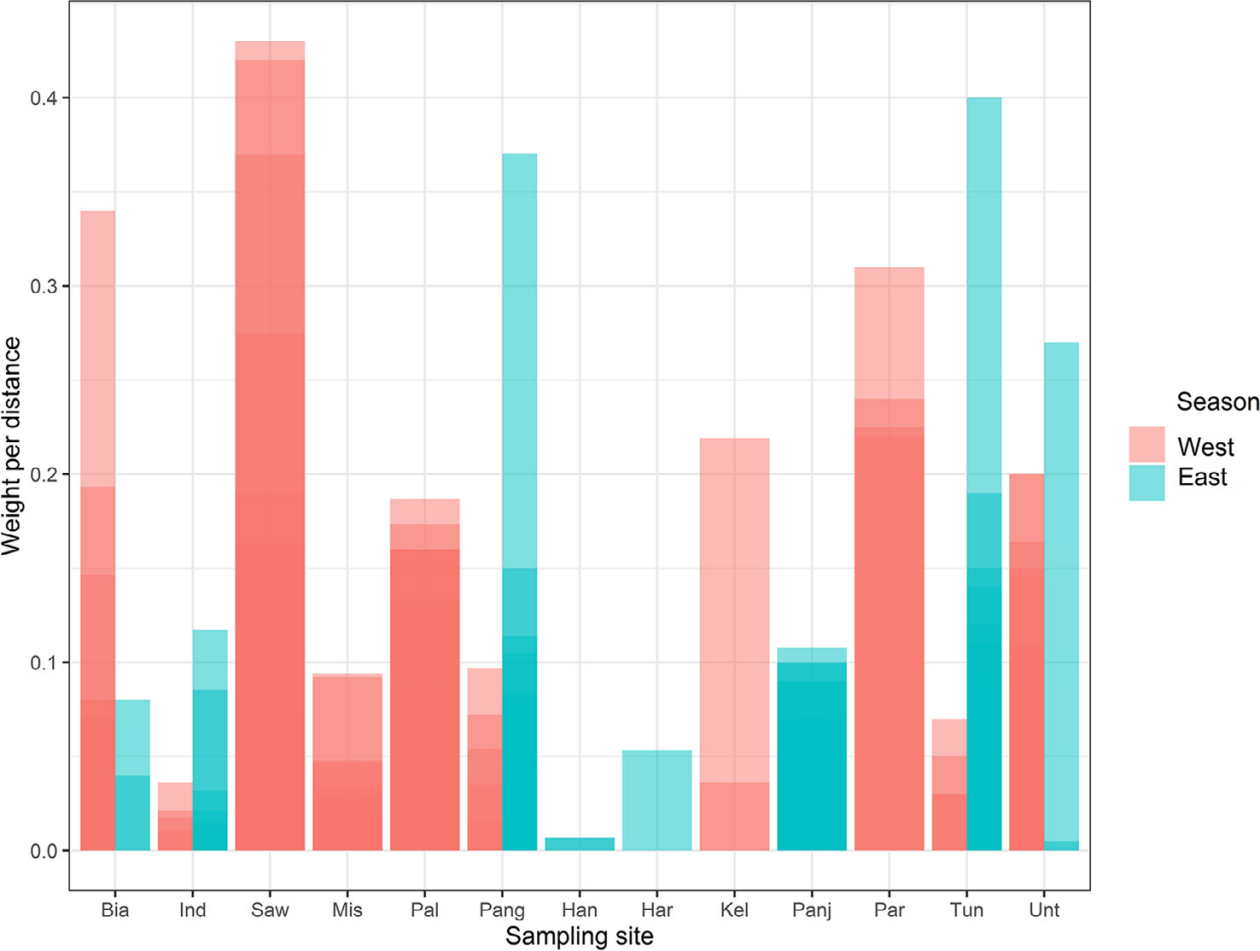
Seasonal distribution of MD in sampling site. Seasons are divided by north-west monsoon (DJF) and south-east monsoon (JJA) with transition monsoon included in each category.

The weights of solid wastes were higher in the stations located in the east side than the west of the Indramayu, Pangandaran, Tunda, and Untung Jawa sampling stations. Moreover, the waste weight ratio between the Biawak and Indramayu showed an inversely proportional relationship even though they were both in North Java but differed between the two seasons. The highest waste ratio in the west station was observed in the Savu Sea, and the lowest is in Indramayu. The above results revealed a correlation relationship with the variables of the ocean currents. Furthermore, the highest waste ratio was in the Australian Monsoon (Jun, Jul, Aug) of Tunda, Pangandaran, Untung Jawa, Indramayu, Panjang Island, Biawak, Harapan Island, and Handeleum, respectively (Fig. 5).

## 2 Experimental Design, Materials and Methods

### 2.1 Sampling method

The sampling method used in this research is based on International Coastal Cleanup (ICC) form developed by Ocean Conservancy International (https://oceanconservancy.org/wp-content/uploads/2017/04/OC-DataCards_volunteerFINAL_ENG.pdf). The line in Transect (LIT) method was used between 100 m parallel to the coastline [4]. All debris > 2.5 cm (macro debris) were collected, categorized, counted, and weighted. These methods can also be combined with visual counting (naked eye). The items were sorted into categories according to the form (A. Most Likely Found Items, B. Fishing Gears, C. Packaging Materials, D. Personal Hygiene, E. Other Trash, and F. Tiny Trash).

**Fig. 6:**
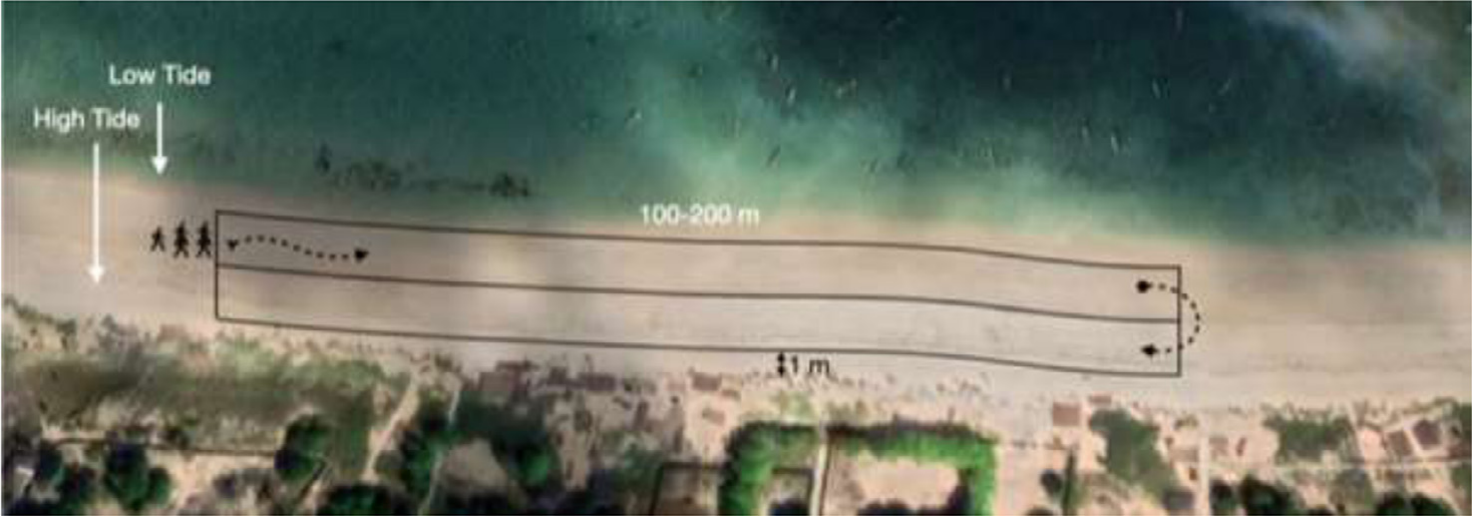
Illustrated MD collection Method in the coastal area. Transect made in an intertidal line with a transect length of 100 m with a buffer as far as 5 m from the transect line. One person held a GPS to mark the position of debris (1), two other persons collected the debris (2), and the last person collected the debris left uncollected (4).

Technically, the observers comprised of three to five persons for 2–3 h of sampling (Fig. 6). The sampling area is 1 square meter between the coastal vegetation and the lowest tide [5]. 1 to 2 people were tasked with the responsibility of mapping the waste using GPS. Then two people collected debris and gathered them in the trash bags. The last person was checking for any trash left along the transect line. Then, the debris was separated and classified according to the designed sheet. In this method, an ocean conservancy sheet is used.

Furthermore, the debris was later weighed. In some cases, the garbage taken from the beach was still wet, so the debris was first allowed to dry. This was done to avoid errors in the weight of the waste. The datasheet and pen were used to record the information provided by sampling equipment, maps, bucket, scales, measuring tape, hand-held GPS, camera, and bags/containers to collect debris. For safety reasons, safety shoes, first aid, and work gloves were used.

Debris that has been collected is later categorized, counted, and weighted. The density and quantity were calculated from each station using statistical analysis. The weight of the debris was divided by the area to obtain debris weight per square meter.

### 2.2 Quality control

Quality control (QC) methods is a technique deployed to test the quality of acquired data. The best method to ensure the acquisition of good quality data is that the method and procedure for data collection should be simulated before sampling [6]. Implementation of QC should be considered before sampling activities, during the sampling, and analysis. First, to acquire quality data, every team member would be briefed about the form sheet, and the expert should ensure the observer is easy to understand. Furthermore, all the observers should do simulation and the expert should ensure data has good quality. During the sampling collection, one expert should follow the observers. In many sampling collection, most of the observer is the same person in order to reduce sampling erors. Sometimes, debris in the beaches was mixed with water or other materials, and it had to be dried before weighing in order to reduce data errors. The expert should carefully read the form sheet and sign if it is already satisfied. Furthermore, comparative data collection was also done by comparing data in several places.

## Supplementary Information

The data is stored in https://data.mendeley.com/datasets/r3y43cdd3x/2.

## CRediT Author Statement

**Ibnu Faizal:** Conceptualization, Writing – original draft; **Zuzy Anna:** Funding acquisition, Conceptualization; **Sanny T. Utami:** Validation, Writing – review & editing; **Putri G. Mulyani:** Data curation; **Noir P. Purba:** Writing – original draft, Methodology.

## Declaration of Competing Interest

The authors declare that there are no known competing financial interests or personal relationships that could have influenced the work reported in this paper.

## Appendix Supplementary Materials

Image, application 1

Image, application 2
