# Supplementary material for: Baseline data of marine debris in the Indonesia beaches
Source: Data Brief. 2022 Jan 25;41:107871. doi: 10.1016/j.dib.2022.107871 (PMC8841578; doi:10.1016/j.dib.2022.107871)
Supplement: Supplementary file 2 [file mmc2.pdf]

VOLUNTEER
OCEAN TRASH DATA FORM

Ocean Conservancy

Ocean and waterway trash ranks as one of the most serious pollution problems choking our planet. Far more than an eyesore, a rising tide of marine debris threatens human health, wildlife, communities and economies around the world. The ocean faces many challenges, but trash should not be one of them. Ocean trash is entirely preventable, and data you collect are part of the solution. The International Coastal Cleanup is the world's largest volunteer effort on behalf of ocean and waterway health.

HERE IS HOW IT WORKS:

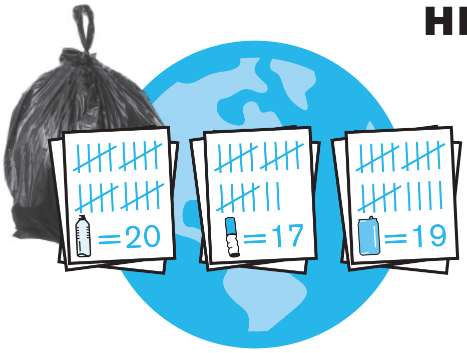

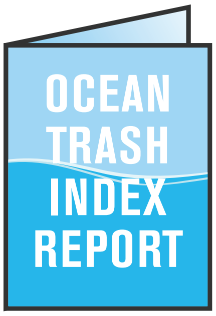

1 CLEAN UP TRASH & COLLECT DATA

3 PUBLISH RESULTS

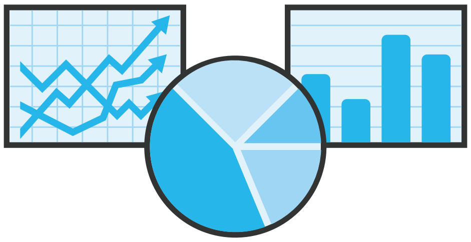

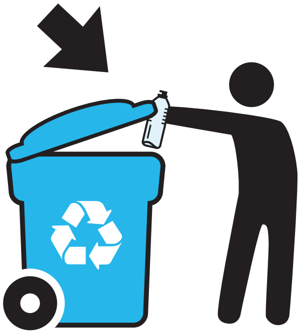

2 ORGANIZE & ANALYZE DATA

4 REDUCE OUR IMPACT

SITE INFORMATION:

Cleanup Site Name: ____________________

State or Province: ____________________ Zone or County: ____________________

Country: ____________________ Nearest Crossroad or Landmark: ____________________

NUMBER OF VOLUNTEERS WORKING ON THIS CARD:

adults ______ children (under 12) ______

MOST UNUSUAL ITEM COLLECTED:

____

TYPE OF CLEANUP:

Land: ☐ Underwater: ☐ Watercraft: ☐

Make positive changes every day by downloading our free mobile app, **RIPPL**

OCEAN CONSERVANCY'S International Coastal Cleanup®

Please return this form to your area coordinator.
If you are unable to do so, please mail or email it to:

Ocean Conservancy
Attn: International Coastal Cleanup
1300 19th Street, NW, 8th Floor
Washington, DC 20036
cleanup@oceanconservancy.org

Trash Free Seas: www.oceanconservancy.org/cleanup
Be a Green Boater: www.oceanconservancy.org/do-your-part/green-boating
Sponsors: www.oceanconservancy.org/cleanupsponsors

TRASH COLLECTED

Citizen scientist: Pick up all trash and record all items you find below. No matter how small the items, the data you collect are important for Trash Free Seas.®

EXAMPLE:	TOTAL #
Plastic Bags: ~~||||~~ |||	= 8

Please DO NOT use words or check marks. Only **numbers** are useful data.

MOST LIKELY TO FIND ITEMS:

Item	TOTAL #
Cigarette Butts:	=
Food Wrappers (candy, chips, etc.):	=
Take Out/Away Containers (Plastic):	=
Take Out/Away Containers (Foam):	=
Bottle Caps (Plastic)	=
Bottle Caps (Metal)	=
Lids (Plastic) :	=
Straws/Stirrers:	=
Forks, Knives, Spoons:	=
Beverage Bottles (Plastic):	=
Beverage Bottles (Glass):	=
Beverage Cans:	=
Grocery Bags (Plastic):	=
Other Plastic Bags:	=
Paper Bags:	=
Cups & Plates (Paper):	=
Cups & Plates (Plastic):	=
Cups & Plates (Foam):	=

FISHING GEAR:

Item	TOTAL #
Fishing Buoys, Pots & Traps:	=
Fishing Net & Pieces:	=
Rope (1 yard/meter = 1 piece):	=
Fishing Line (1 yard/meter = 1 piece):	=

OTHER TRASH:

Item	TOTAL #
Appliances (refrigerators, washers, etc.):	=
Balloons:	=
Cigar Tips:	=
Cigarette Lighters:	=
Construction Materials:	=
Fireworks:	=
Tires:	=

PACKAGING MATERIALS:

Item	TOTAL #
6-Pack Holders	=
Other Plastic/Foam Packaging:	=
Other Plastic Bottles (oil, bleach, etc.):	=
Strapping Bands:	=
Tobacco Packaging/Wrap:	=

PERSONAL HYGIENE:

Item	TOTAL #
Condoms:	=
Diapers:	=
Syringes:	=
Tampons/Tampon Applicators:	=

TINY TRASH LESS THAN 2.5CM:

Item	TOTAL #
Foam Pieces	=
Glass Pieces	=
Plastic Pieces	=

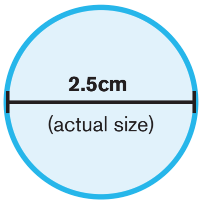

DEAD/INJURED ANIMAL	STATUS	ENTANGLED	TYPE OF ENTANGLEMENT ITEM
	**Dead** or **Injured**	**Yes** or **No**	

ITEMS OF LOCAL CONCERN:

1.
2.
3.

CLEANUP SUMMARY (circle units)

Number of Trash Bags Filled: ____ **Weight of Trash Collected:** ____ lbs/kgs **Distance Cleaned:** ____ miles/km
